# Plant–animal worms round themselves up in circular mills on the beach

**DOI:** 10.1098/rsos.180665

**Published:** 2018-07-25

**Authors:** Ana B. Sendova-Franks, Nigel R. Franks, Alan Worley

**Affiliations:** 1 Department of Engineering Design and Mathematics, UWE Bristol, Frenchay Campus, Coldharbour Lane, Bristol BS16 1QY, UK; 2 School of Biological Sciences, University of Bristol, 24 Tyndall Avenue, Bristol BS8 1TQ, UK

**Keywords:** circular mills, collective motion, collective behaviour

## Abstract

Collective motion is a fascinating and intensely studied manifestation of collective behaviour. Circular milling is an impressive example. It occurs in fishes, processionary caterpillars and army ants, among others. Its adaptive significance, however, is not yet well understood. Recently, we demonstrated experimentally circular milling in the marine plant–animal worm *Symsagittifera roscoffensis*. We hypothesized that its function is to gather the worms and facilitate the dense films they form on the beach to promote the photosynthesis of their symbiotic algae. Here, we report for the first time, to our knowledge, the occurrence of *S. roscoffensis* circular mills in nature and show that it is by no means rare. The size and behaviour of circular mills in their natural environment is compatible with our earlier experimental results. This makes *S. roscoffensis* a good study system for understanding the proximate and ultimate mechanisms of circular milling.

## 1. Introduction

The adaptive significance and underlying mechanisms of collective motion are fundamental to understanding collective behaviour [1–6]. Circular milling is a case in point: it occurs across taxa [7–12], but its function is still not fully understood [7,12].

Recently, we reported for the first time, to our knowledge, circular mills in the plant–animal worm *Symsagittifera roscoffensis* (Ludwig Von Graaf 1891) [12]. These worms live in the high intertidal zone of the East Atlantic and subsist on nutrients produced in symbiosis with the photosynthesizing alga *Platymonas convolutae* [13]. Between April and September, they appear in shallow pools of seeping seawater on the sand as it is uncovered by the receding tide and disappear as quickly back into the sand when the tide comes in [14].

We demonstrated that these plant–animals are social: they interact more often than expected by chance and with increasing density transition from flotillas, comprising only a few individuals swimming tightly in the same direction, to circular mills comprising many more worms moving synchronously clockwise or anti-clockwise [12]. We hypothesized that the circular mills gather up the worms into dense films that, through the protection of a mucous cover, maximize the absorption of light for the photosynthesis by the symbiotic algae [12,14].

Our results were obtained under experimental conditions, in Petri dishes of seawater with different densities of *S. roscoffensis* worms. Although the importance of the phenomenon is clear, up to now, there has been no evidence that the circular milling by *S. roscoffensis* occurs in nature. Indeed, some scientists doubted it does [15]. Here, we provide such evidence.

We show that circular mills of *S. roscoffensis* occur naturally on the intertidal sand and that such occurrences are by no means rare events. For five consecutive days, we photographed the immediate surroundings of 10 individually marked boulders along a small section of the high end of the intertidal beach on the north coast of Guernsey. Our aim was to gauge the dynamics of the pattern of *S. roscoffensis* patches between successive low tides and establish whether any circular mills would occur as part of such dynamics during our daily scan over the observation period.

## 2. Material and methods

**Figure 1.**
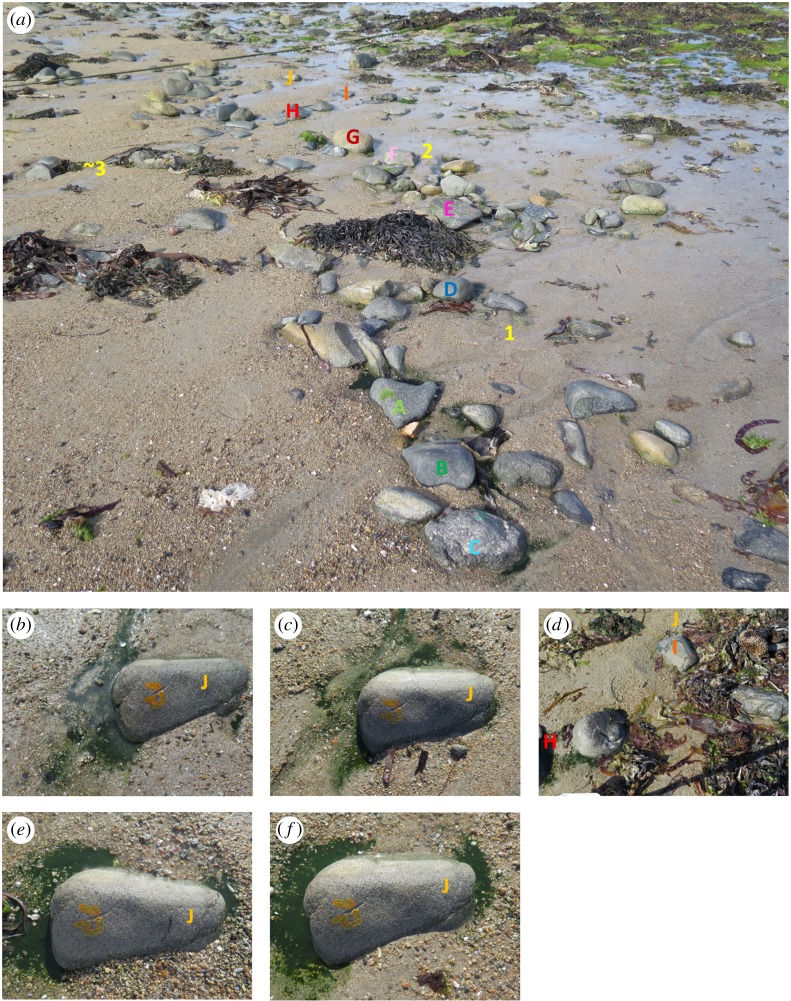
The boulders used for sampling. (*a*) An overview of all 10 individually marked boulders (A–J) on the beach on 13 June 2017, 112 min after low tide; the letter colours correspond to the colour of the varnish used to mark the respective boulder; the yellow numbers 1–3 indicate the positions of the observed circular mills; the patches of *S. roscoffensis* can be seen clearly around the proximate boulders, A–C, but all boulders had worm patches around them on each of the 5 days of observation. The five photographs of boulder J. (*b*) 10 June, 15.45 (95 min after low tide). (*c*) 11 June, 17.24 (164 min). (*d*) 12 June, 17.25 (125 min), boulder J was completely covered by seaweed and was not visible, it is in the area marked with J north of boulder I, which is in the field of view together with part of boulder H. (*e*) 13 June, 17.28 (98 min). (*f*) 14 June, 16.46 (26 min), note the difference between 13 and 14 June in the sand composition to the south of the boulder.

The observations took place between 10 and 14 June 2017 on a beach on the north coast of Guernsey. Once on each of the 5 days, we photographed the patches of *S. roscoffensis* in the immediate vicinity of 10 boulders marked individually with the letters A–J in a unique colour of varnish. The largest dimension of each boulder was approximately 30 cm and they all retained their positions throughout the study period. The boulders were chosen to form a transect approximately orthogonal to the shore, situated between two mooring ropes approximately 10 m apart (figure 1*a*).

The transect was approximately 5 m from the high-water mark and approximately 70 m from the low-water mark. The beach had a complex structure of roughly four zones. Starting from the high end, these comprised: (i) approximately 5 m of boulders; (ii) approximately 5 m of a mixture of quickly drying sand with a few stationary boulders of similar size to those in (i); (iii) approximately 40 m of a mixture of more such boulders with sand, seeping water, algae and seaweed; and (iv) approximately 40 m of mostly wet sand with fewer boulders, algae or seaweed. The high-water mark was in the bottom half of the first zone made up solely of boulders. Our transect was near the top end of the third zone.

The worm patches started to appear very quickly approximately 160 min after the high tide and approximately 5–10 min after no waves of the receding tide reached them any more (11 June 2017, low tide at 08.30, first worm patches appeared at 11.05–11.10). Circular mills were observed in little pools of water approximately 5 mm deep.

The daytime low tides on 10–14 June 2017 were at: 14.12, 14.45, 15.17, 15.49 and 16.22, respectively. Photographs of the transect were taken starting from boulder A and moving towards boulder J on each of 10–14 June 2017 between 15.00 and 15.45, 17.15 and 17.30, 17.00 and 17.50, 17.15 and 17.45, and 16.30 and 16.45, respectively. The sampling durations were very similar even though the visits to the beach were longer on 10 and 12 June. The former was the day when the boulders were marked and the latter was the only day when we used a tripod to mount the Canon G16 camera we used for recording.

## 3. Results

The immediate environment around each boulder changed between days and with the time elapsed since the last low tide. The daily changes were predominantly in the micro-landscape of shallow hollows and channels that could hold the seeping seawater and provide a sunning opportunity for the worms. By contrast, the seaweed brought by the latest high tide could hide a boulder and its immediate surroundings completely thus preventing the exposure of the photosynthesizing worms to the light (figure 1*b–f*; electronic supplementary material, S1–S9). As the time since the low tide increased, the shallow pools of water round the boulders tended to decrease in size or disappear altogether. Given all these sources of change, it is not surprising that the pattern of *S. roscoffensis* patches also changed (figure 1*b–f*; electronic supplementary material S1–S9). The biofilms of worms thrive in shallow pools of water but the worms bury themselves in the sand if the water evaporates. This prompts the question of whether the worms need the shallow water to move to a more promising location or whether they can displace themselves in the sand.

**Figure 2.**
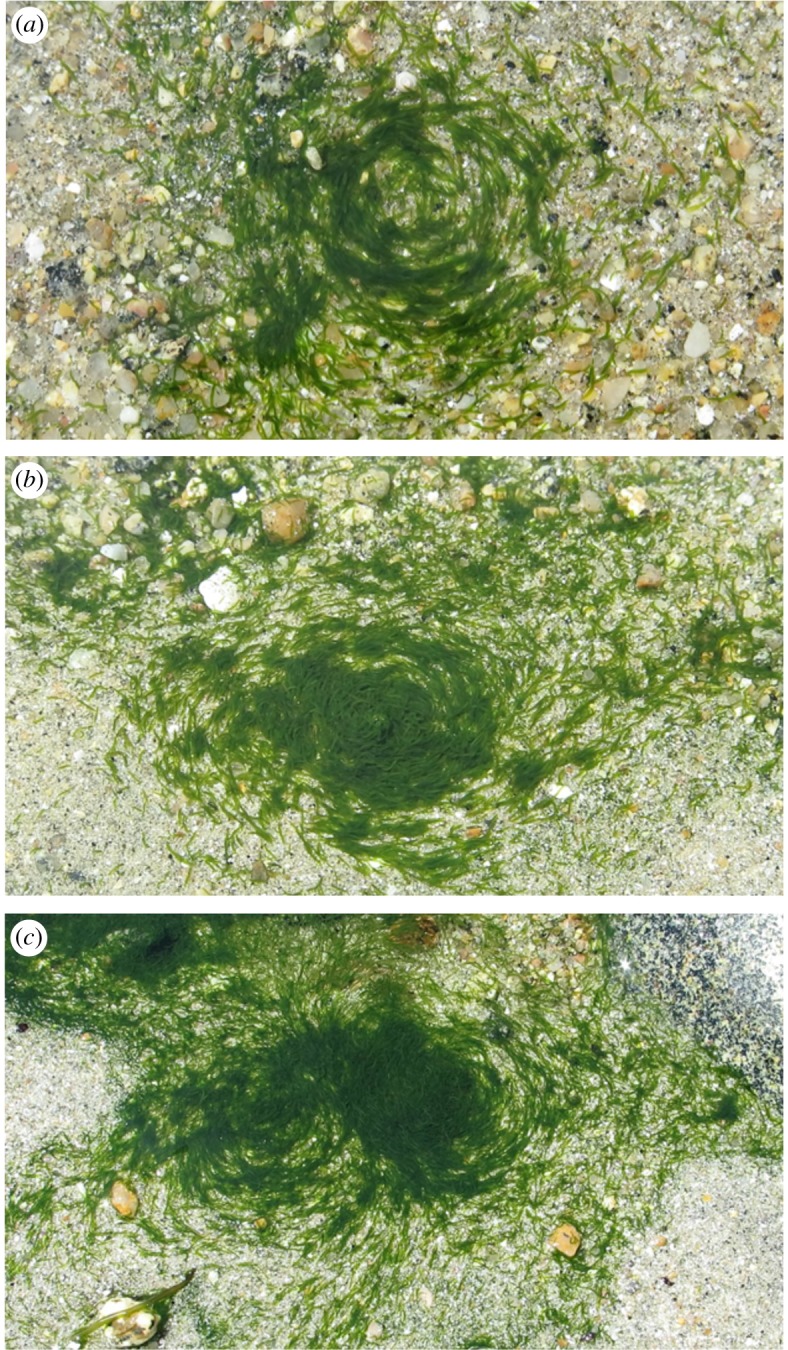
The three circular mills of *S. roscoffensis* filmed on the beach. (*a*) Circular mill number 1 was observed near boulder D on 10 June 2017 (figure 1*a*). (*b*) Circular mill number 2 was observed near boulder F on 10 June (figure 1*a*). Both circular mills 1 and 2 were moving clockwise. (*c*) Twin circular mill number 3 was observed on 14 June up the beach from boulder H (figure 1*a*) and consisted of a right mill going clockwise and a left mill going anti-clockwise. Each individual mill was between 20 and 30 mm in diameter. The depth of water was approximately 5 mm. The boundaries of the water pools were not critical for circular mill formation because the smallest pool dimension was larger than the mill diameter (electronic supplementary material, videos S1–S3).

Over the 5 days, we observed altogether four circular mills. Numbers 1 and 2 were photographed and filmed on 10 June during the sampling of the transect between 15.00 and 15.45 close to boulders D and F, respectively (figure 2*a,b*; electronic supplementary material, videos S1 and S2). We encountered number 3 by chance on 14 June at 13.30 (before the sampling period from 16.30 to 16.45) approximately 2 m up the beach from boulder H (figure 2*c*) while collecting seawater and worms for another study and returned immediately to photograph and film it. The two twins in this mill (figure 2*c*; electronic supplementary material, video S3) rotate in opposite directions like meshing gears. As we have shown earlier [12], one of the defining features of social behaviour in these worms at lower densities is to swim in parallel. In the context of twin circular mills, this could only be achieved if they rotate in opposite directions. Circular mill number 4 was also encountered by chance on 10 June at 16.00 (after the sampling period) higher on the beach within the same sector towards boulder J. It was not photographed or filmed owing to time constraints.

There were patches of moving worms that were not forming circular mills (electronic supplementary material, figure S10). It seems that on the beach, density is critical as it is in the laboratory [12].

## 4. Discussion

The beach environment for *S. roscoffensis* is clearly dynamic and unpredictable. To optimize their chances of forming biofilms to catch vital sunlight in such precarious conditions, the worms are likely to employ movement and aggregation procedures. Circular milling could be one such tactic, acting as a self-organizing windlass dragging in peripheral worms to form high-density biofilms. We hypothesize that the worms are joining a circular track from the outside, while the ones already in the circuit have to move inwards to avoid the newcomers, and eventually the centre grinds to a halt. This individual behavioural rule of joining on the outside and moving inward means that the mills would initially have hollow centres and then, if there are sufficient worms, fill up. This scenario is supported by the morphologies of circular mills we have recorded under experimental conditions ([12]; electronic supplementary material, figures S11 and S12). The logical consequence of this process is the prediction that the worms near the centre make many more changes of direction as they have to avoid more and more neighbours. Eventually, they need to stop moving or dive to get out of the way.

We found that the smaller circular mills have an empty core (figure 2*a*) and the larger circular mills have a filled core (figure 2*b,c*). This is evidence in support of the process underlying circular milling proposed above and suggests that circular mills do allow worms to achieve higher density prior to forming a biofilm. Our result that circular mills occur frequently lends further support to the hypothesis that they are adaptive.

Having established that circular mills in *S. roscoffensis* are not an artefact of Petri dishes, further experimentation is justified. Here is a case where organisms readily form circular mills both in the field and under experimental conditions. This identifies plant–animal worms as a beautiful model system for studying circular milling.

Across taxa, some circular mills are adaptive (e.g. shoals of fishes, because fishes always move [11]) and some are likely to be maladaptive (e.g. army ants [7]). More detailed analysis of the process in *S. roscoffensis* is required to demonstrate that the circular mills they form are an adaptive feature of their behaviour and ecology. For example, future studies need to establish whether the circular mill travels as well as spins. In other words, whether it could displace the worms to a different location as well as gather them together.

## Supplementary Material

Supplementary Information

## Data accessibility

The data supporting this article are included in it and the electronic supplementary material.
