# Supplementary material for: Plant–animal worms round themselves up in circular mills on the beach
Source: R Soc Open Sci. 2018 Jul 25;5(7):180665. doi: 10.1098/rsos.180665 (PMC6083728; doi:10.1098/rsos.180665)
Supplement: Supplementary Information [file rsos180665supp1.docx]

SUPPLEMENTARY INFORMATION

**Plant-Animal Worms Round Themselves up in Circular Mills on the Beach**

Ana. B. Sendova-Franks, Nigel R. Franks and Alan Worley


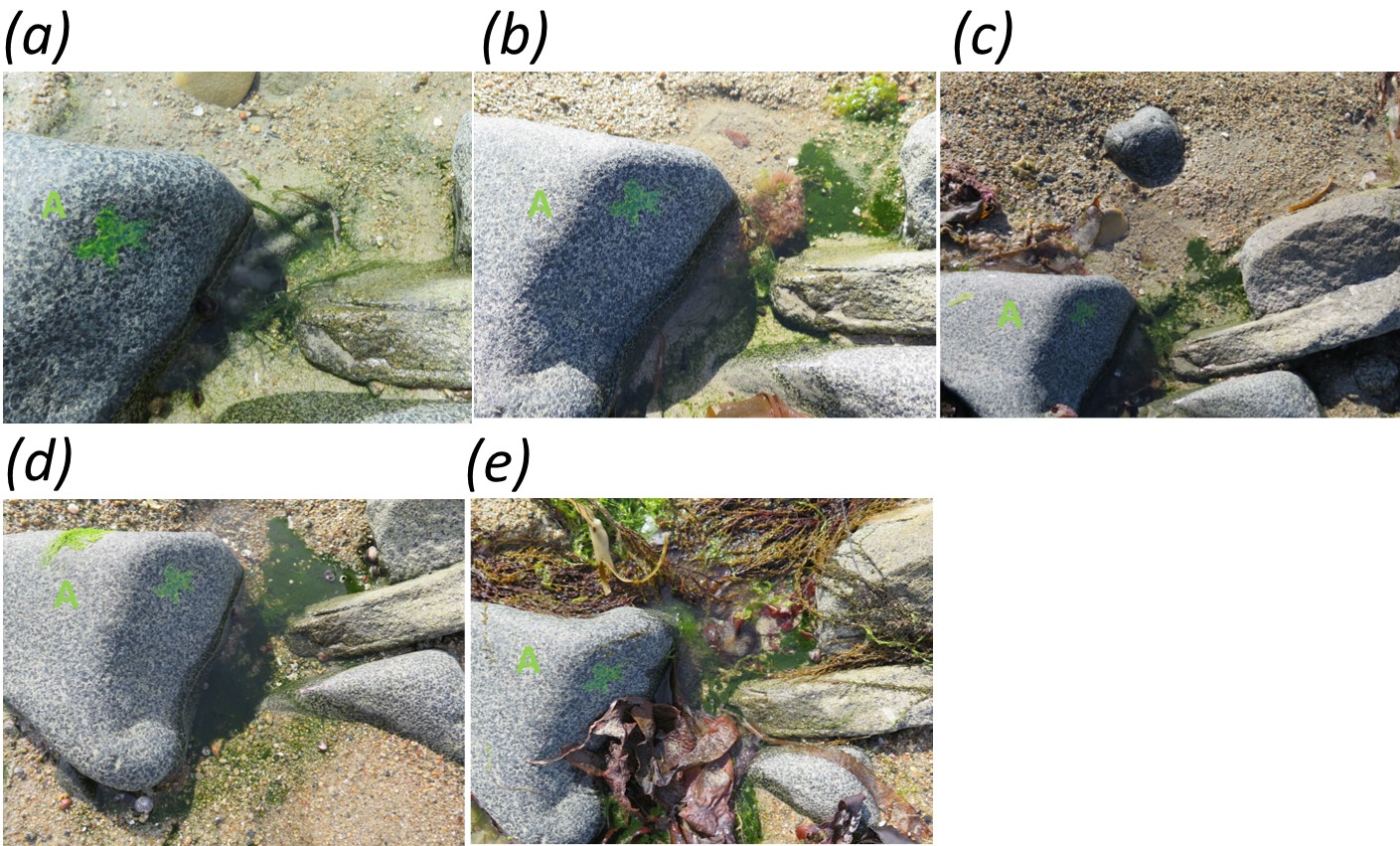


**Figure S1.** The five photographs of boulder A: *(a)* 10^th^ June, 15:10 (60min after low tide). *(b)* 11^th^ June, 17:15 (155min). *(c)* 12^th^ June, 17:02 (102min). *(d)* 13^th^ June, 17:19 (89min). *(e)* 14^th^ June, 16:39 (19min).


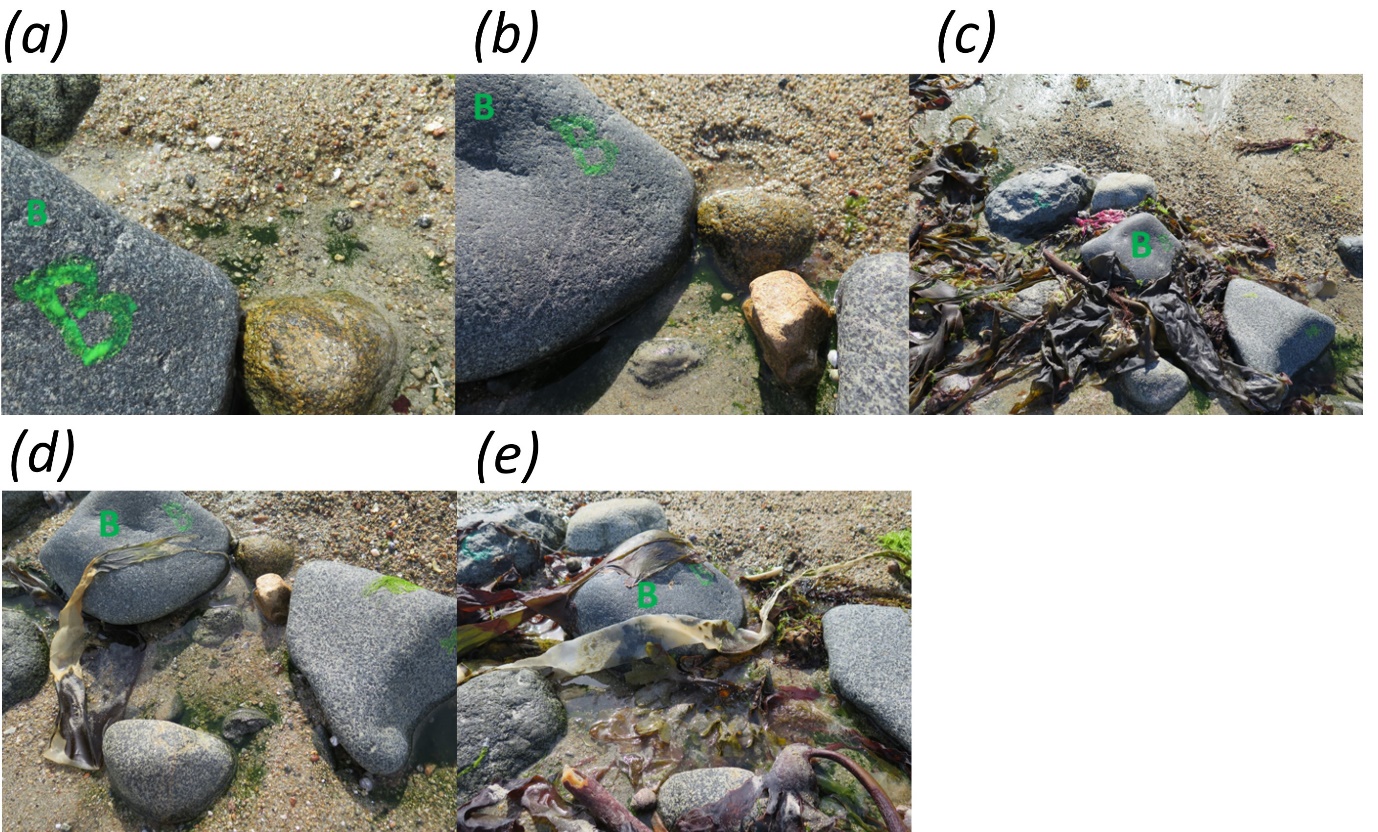
**Figure S2.** The five photographs of boulder B: *(a)* 10^th^ June, 15:12 (62min after low tide). *(b)* 11^th^ June, 17:16 (156min). *(c)* 12^th^ June, 17:03 (103min). *(d)* 13^th^ June, 17:20 (90min). *(e)* 14^th^ June, 16:39 (19min).


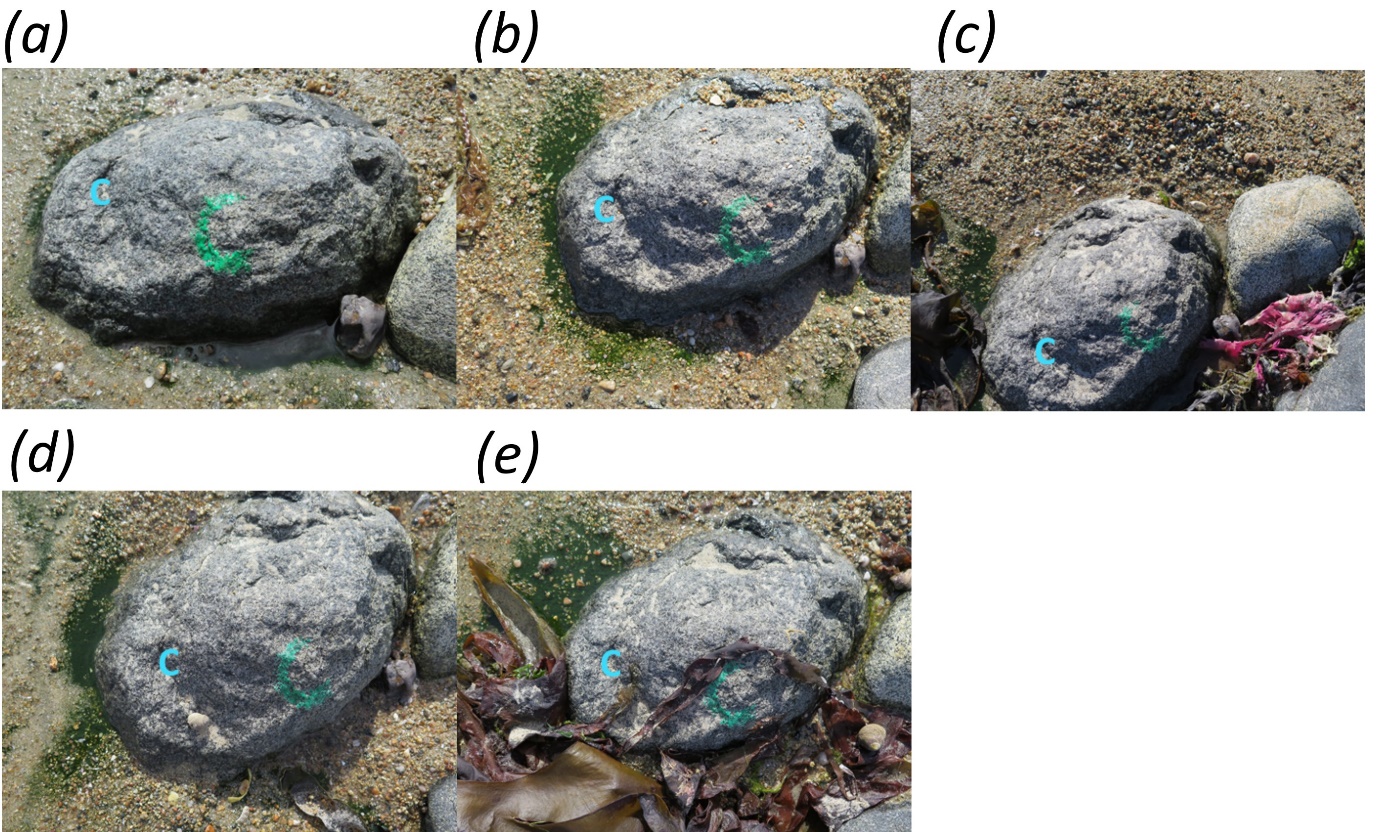


**Figure S3.** The five photographs of boulder C: *(a)* 10^th^ June, 15:14 (64min after low tide). *(b)* 11^th^ June, 17:16 (156min). *(c)* 12^th^ June, 17:04 (104min). *(d)* 13^th^ June, 17:21 (91min). *(e)* 14^th^ June, 16:40 (20min).


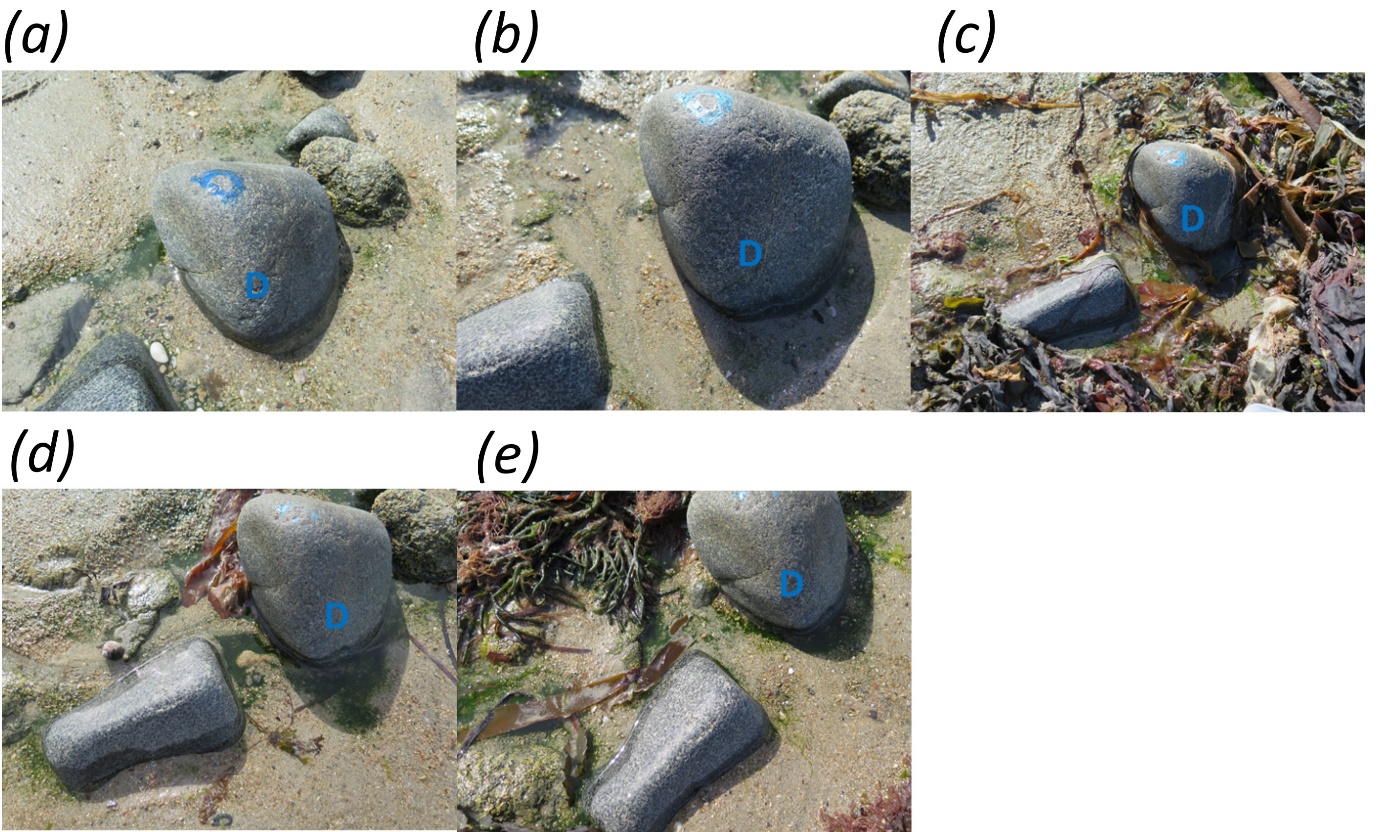


**Figure S4.** The five photographs of boulder D: *(a)* 10^th^ June, 15:16 (66min after low tide). *(b)* 11^th^ June, 17:17 (157min). *(c)* 12^th^ June, 17:10 (110min). *(d)* 13^th^ June, 17:21 (91min). *(e)* 14^th^ June, 16:40 (20min).


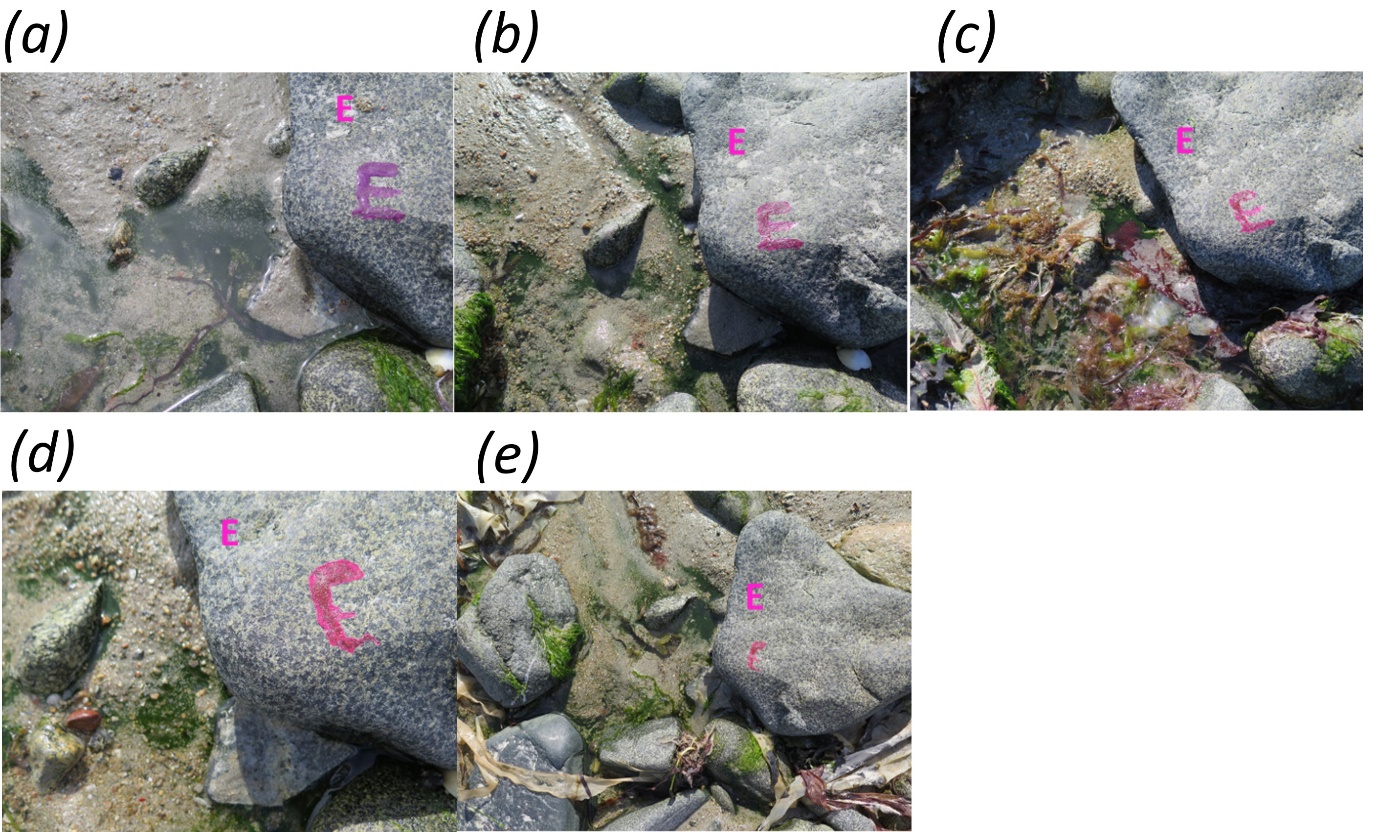


**Figure S5.** The five photographs of boulder E: *(a)* 10^th^ June, 15:32 (82min after low tide). *(b)* 11^th^ June, 17:19 (159min). *(c)* 12^th^ June, 17:12 (112min). *(d)* 13^th^ June, 17:25 (95min). *(e)* 14^th^ June, 16:43 (23min).


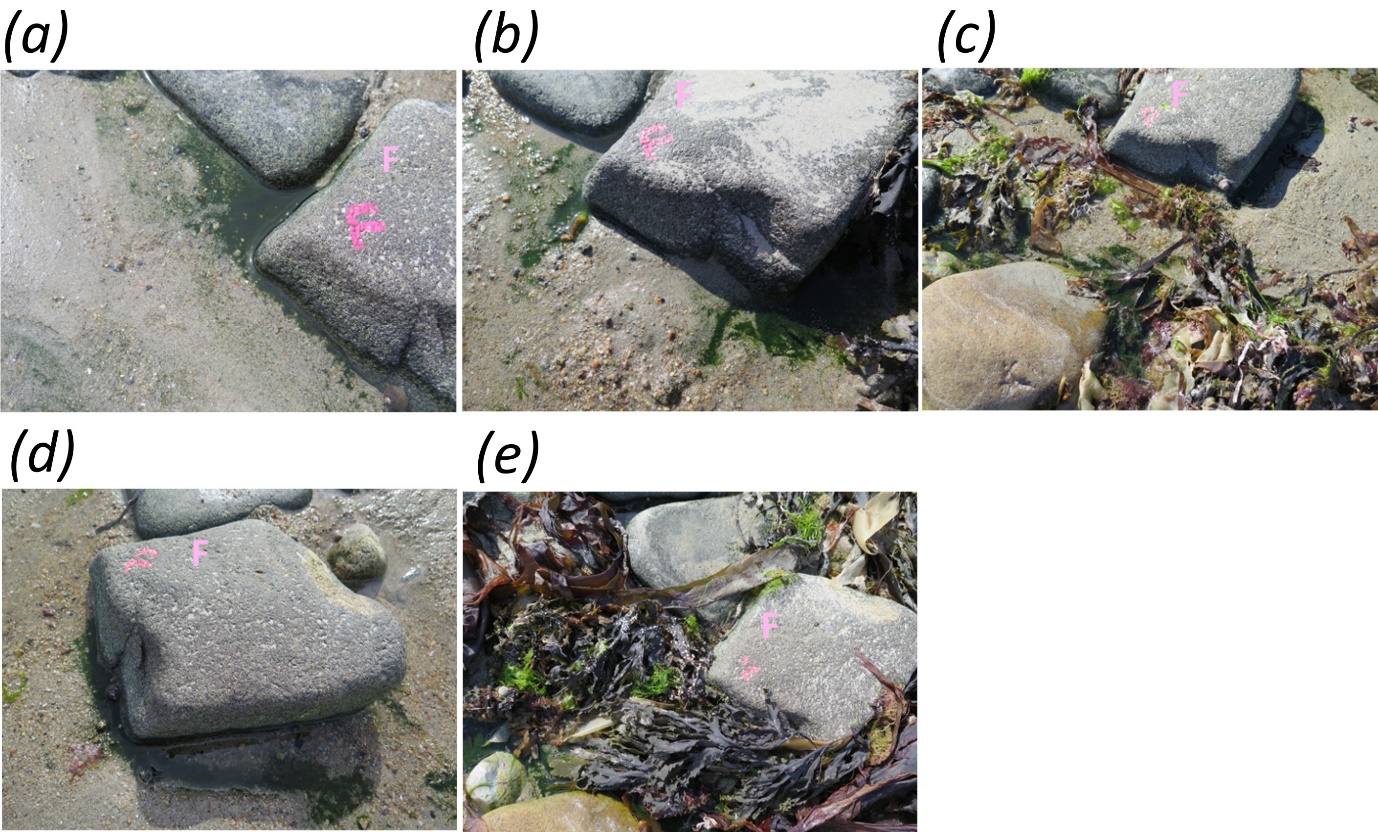


**Figure S6.** The five photographs of boulder F: *(a)* 10^th^ June, 15:33 (83min after low tide). *(b)* 11^th^ June, 17:19 (159min). *(c)* 12^th^ June, 17:14 (114min). *(d)* 13^th^ June, 17:26 (96min). *(e)* 14^th^ June, 16:43 (23min).


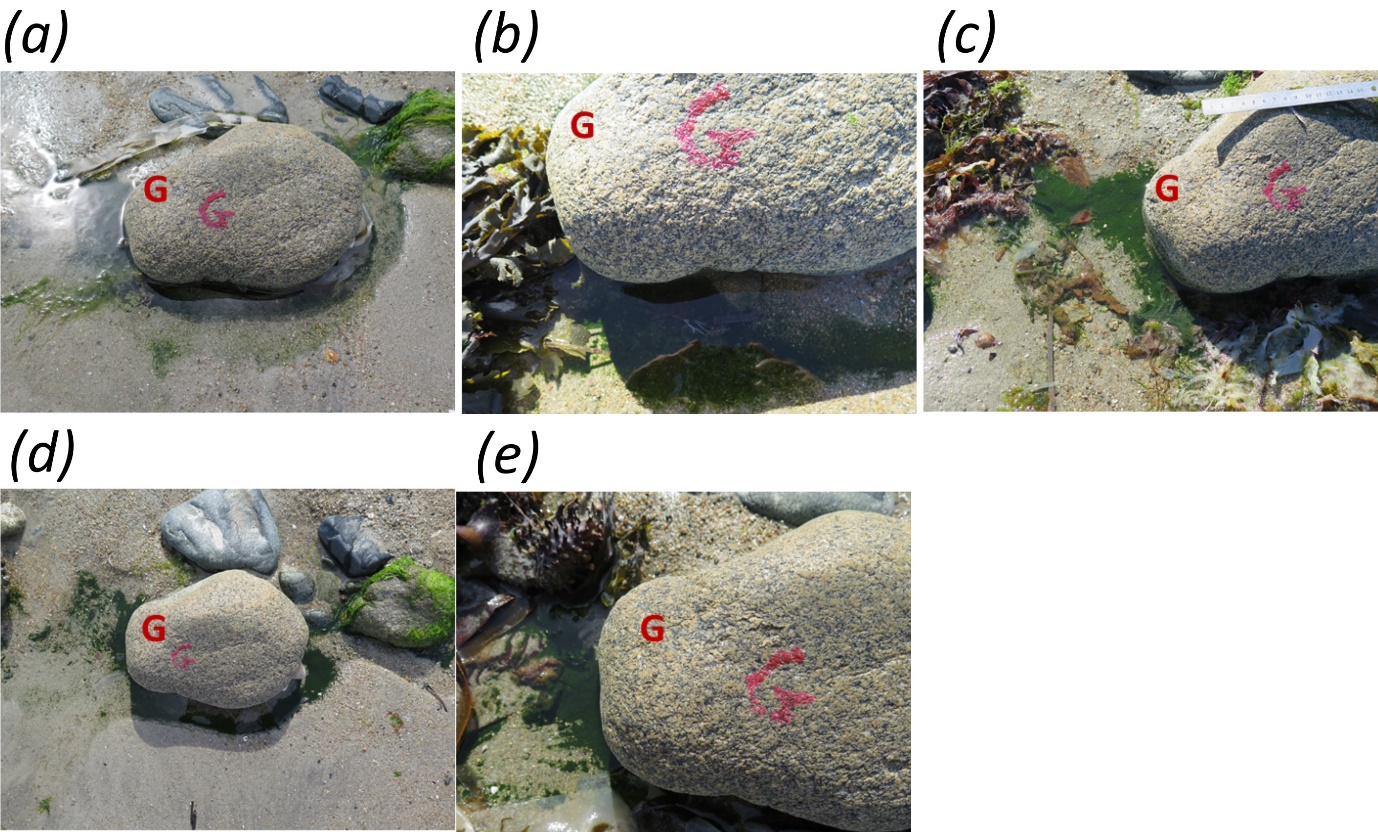


**Figure S7.** The five photographs of boulder G: *(a)* 10^th^ June, 15:39 (89min after low tide). *(b)* 11^th^ June, 17:20 (160min). *(c)* 12^th^ June, 17:21 (121min). *(d)* 13^th^ June, 17:27 (97min). *(e)* 14^th^ June, 16:44 (24min).


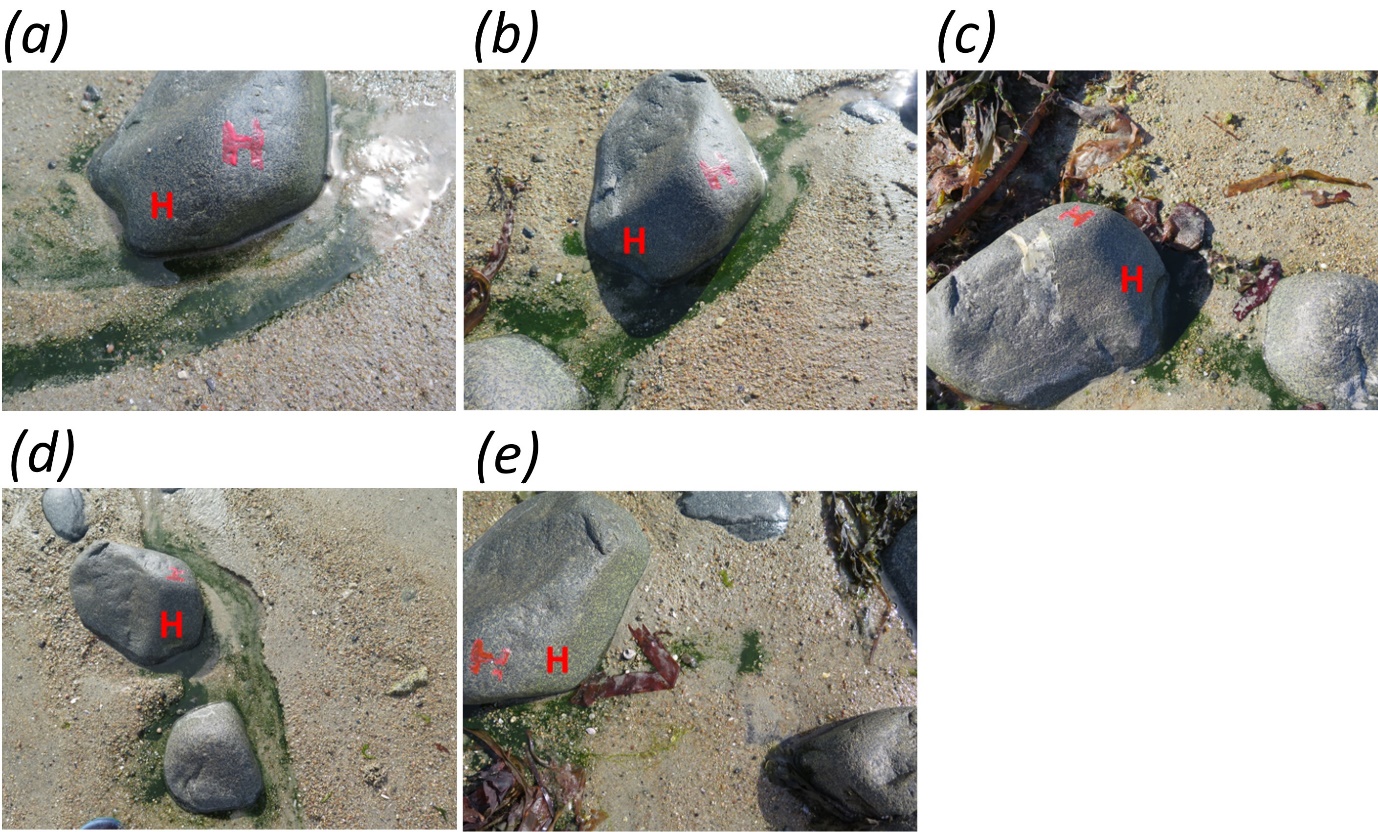


**Figure S8.** The five photographs of boulder H: *(a)* 10^th^ June, 15:42 (92min after low tide). *(b)* 11^th^ June, 17:22 (162min). *(c)* 12^th^ June, 17:24 (124min). *(d)* 13^th^ June, 17:27 (97min). *(e)* 14^th^ June, 16:45 (25min).


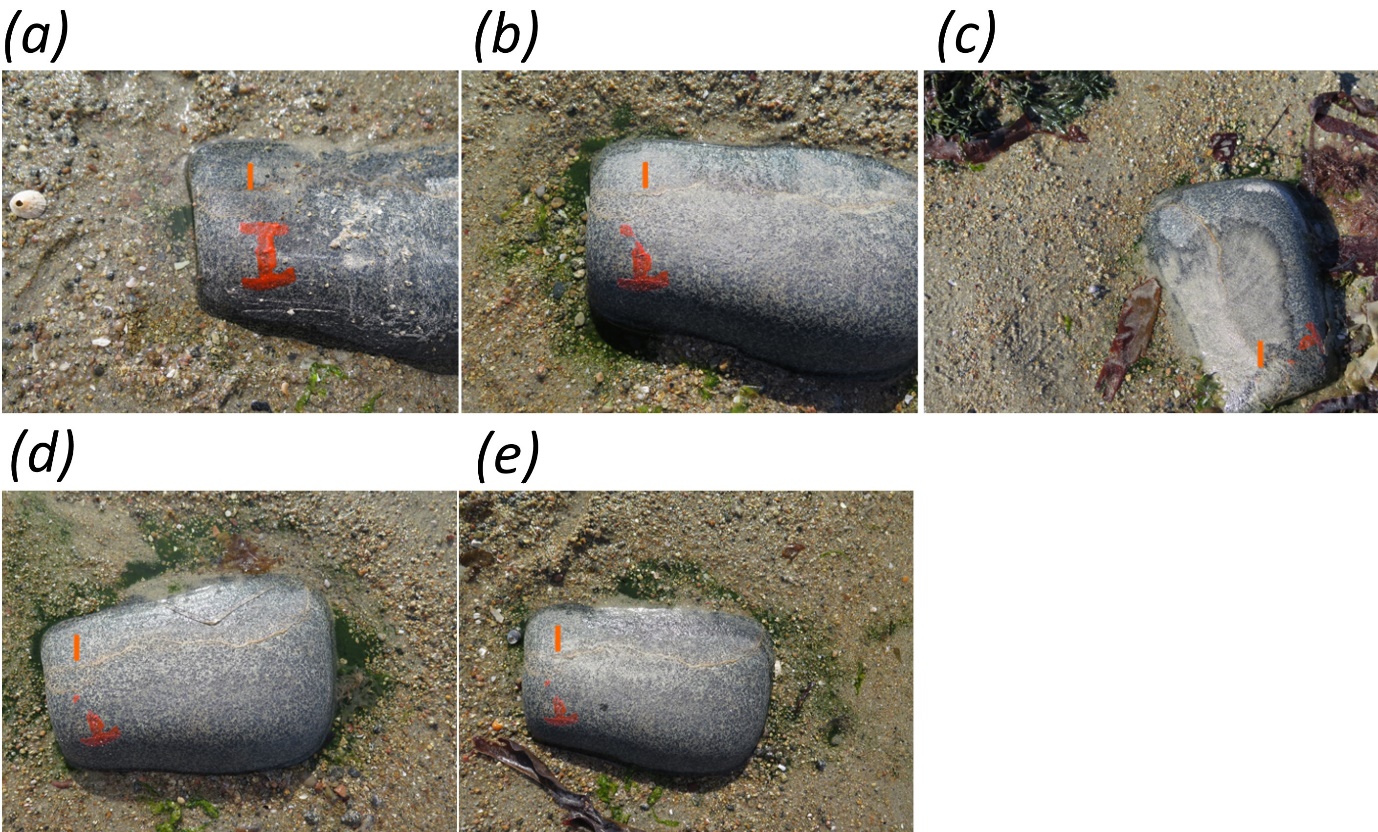


**Figure S9.** The five photographs of boulder I: *(a)* 10^th^ June, 15:43 (93min after low tide). *(b)* 11^th^ June, 17:23 (163min). *(c)* 12^th^ June, 17:26 (126min). *(d)* 13^th^ June, 17:28 (98min). *(e)* 14^th^ June, 16:46 (26min).


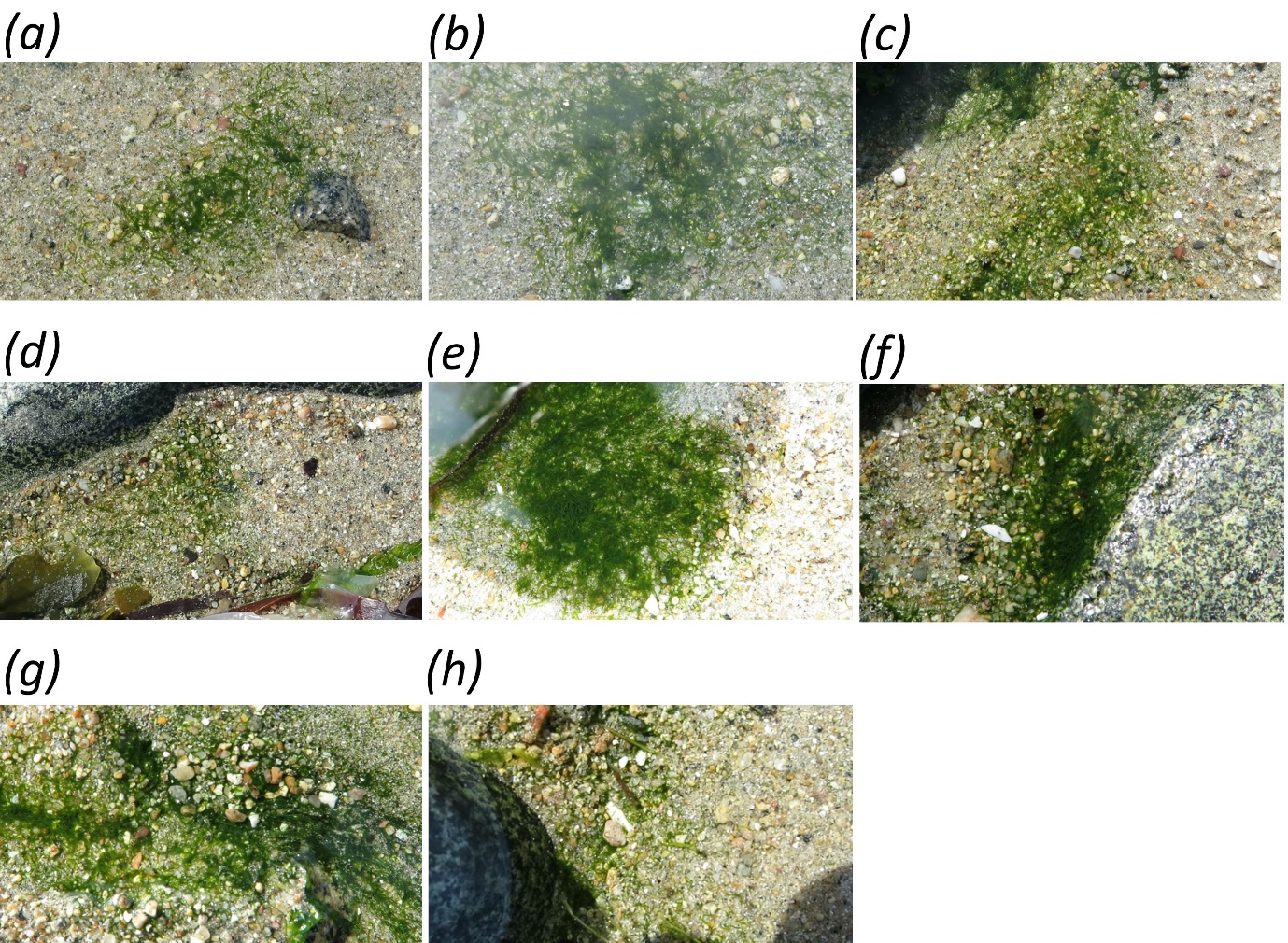


**Figure S10.** Movement but no circular mills: *(a)* 10^th^ June, 15:16 (66min after low tide). *(b)* 10^th^ June, 15:39 (89min). *(c)* 11^th^ June, 17:21 (161min). *(d)* 12^th^ June, 17:08 (108min). *(e)* 13^th^ June, 17:23 (93min). *(f)* 13^th^ June, 17:31 (101min). *(g)* 13^th^ June, 17:34 (104min). *(h)* 14^th^ June, 16:42 (22min).


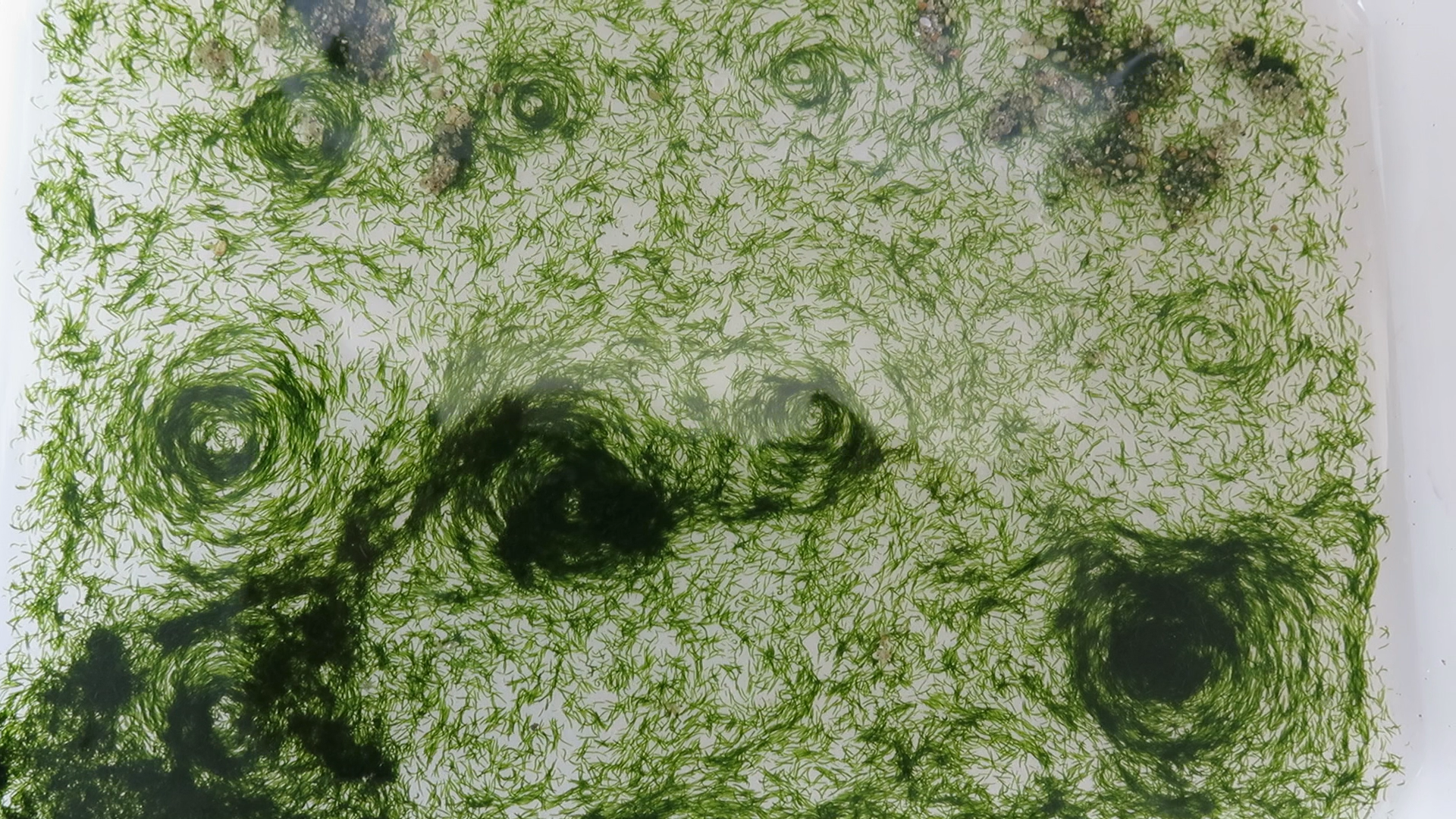


**Figure S11.** Nine circular mills of different density in a tub ~20 x 12cm, 14^th^ June 2016.


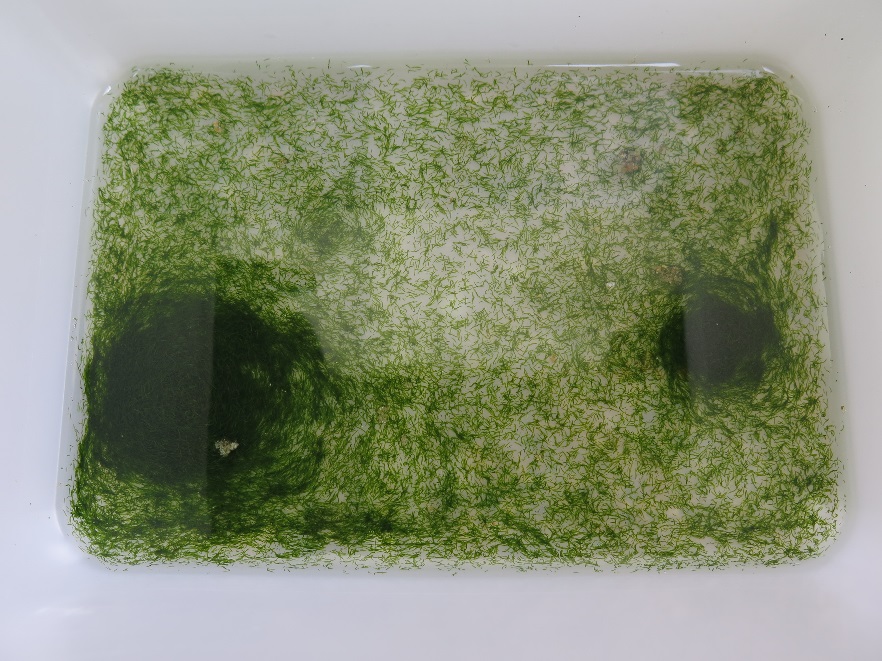


**Figure S12.** Two very dense circular mills in a tub ~20 x 12cm, 15^th^ June 2016.

**Video S1.** Video of circular mill number 1 on the beach near boulder D (figures 2*a*) at 2 x normal speed.

**Video S2.** Video of circular mill number 2 on the beach near boulder F (figures 2*b*) at 2 x normal speed.

**Video S3.** Video of twin circular mill number 3 up the beach from boulder H (figures 2*c*) at 2 x normal speed.
